# A Novel Intravesical Dextrose Injection Improves Lower Urinary Tract Symptoms on Interstitial Cystitis/Bladder Pain Syndrome

**DOI:** 10.3389/fphar.2021.755615

**Published:** 2021-12-15

**Authors:** Chin-Li Chen, Chien-Chang Kao, Ming-Hsin Yang, Gang-Yi Fan, Juin-Hong Cherng, Chih-Wei Tsao, Sheng-Tang Wu, Tai-Lung Cha, En Meng

**Affiliations:** ^1^ Division of Urology, Department of Surgery, Tri-Service General Hospital, National Defense Medical Center, Taipei, Taiwan; ^2^ Graduate Institute of Life Sciences, National Defense Medical Center, Taipei, Taiwan; ^3^ Department and Graduate Institute of Biology and Anatomy, National Defense Medical Center, Taipei, Taiwan; ^4^ Department and Graduate Institute of Biochemistry, National Defense Medical Center, Taipei, Taiwan

**Keywords:** bladder pain syndrome, interstitial cystitis, hyaluronic acid receptor, dextrose prolotherapy, immune system, inflammation

## Abstract

Interstitial cystitis/bladder pain syndrome (IC/BPS) is a painful recurrent condition characterized by the discomfort of the bladder, and current treatment options have limited effectiveness. Prolotherapy is a well-known treatment that involves the injection of non-biologic solutions to reduce pain and/or promote proliferation of soft tissue, and dextrose is the most common injectate. This study investigated the effects of dextrose prolotherapy in a rat model of IC/BPS and patients with IC/BPS. We used cyclophosphamide to induce IC/BPS in rats, and intravesical instillation of 10% dextrose solution was performed. After 1 week, we conducted a urodynamic test, bladder staining, and ECM-related gene expression analysis to examine the treatment’s efficacy. We found that dextrose treatment could recover the instability of the bladder, reduce frequent urination, and improve the glycosaminoglycan layer regeneration and the bladder wall thickness along with a significant intense expression of CD44 receptors. Furthermore, we enrolled 29 IC/BPS patients with previous hyaluronic acid/Botox treatment for more than 6 months with remained unchanged condition. In this study, they received intravesical injections of 10% dextrose solution followed by assessments for up to 12 weeks. Patient characteristics and a 3-day voiding diary before treatment were recorded. Patient responses were examined using IC/BPS-related questionnaires. Moreover, expressions of growth factors and cytokines were analyzed. The results demonstrated that dextrose prolotherapy in patients with IC/BPS reduced the frequency of treatment over time, with the mean number of treatments being 3.03 ± 1.52, and significantly reduced the incidence of nocturia and questionnaire scores associated with symptoms. Dextrose prolotherapy significantly enhanced EGF level and, in contrast, reduced the level of HGF, PIGF-1, and VEGF-D after several weeks following treatment. The cytokine analysis showed that the expressions of IL-12p70 and IL-10 were significantly up-regulated after dextrose prolotherapy in IC/BPS patients. The levels of most growth factors and cytokines in IC/BPS patients had no significant difference and showed a similar tendency as time progressed when compared to healthy controls. Overall, the alteration of growth factors and cytokines exhibited safe treatment and potential stimulation of tissue remodeling. In summary, our study demonstrated that dextrose prolotherapy is a promising treatment strategy for IC/BPS disease management.

## Introduction

Interstitial cystitis/bladder pain syndrome (IC/BPS) is a chronic bladder disorder with unclear etiology characterized with urgency, frequency, and suprapubic discomfort/pain during bladder filling (Chancellor and Yoshimura, 2004). Accumulated evidence showed that IC/BPS is a heterogeneous disorder and is diagnosed after excluding specific lower urinary tract tumors and other diseases. Although many pathogeneses have been suggested, such as post-infectious autoimmune response, mast cell activation, toxin or stress, urothelial dysfunction with increased urothelium permeability, neurogenic inflammation, sensory fiber upregulation, neuropeptide release (P substance), its actual etiology remains unclear (Nickel, 2004).

Currently, no single effective treatment has been recognized for IC/BPS. Oral pentosan polysulfate (Elmiron®) and intravesical dimethyl sulfoxide (DMSO) are two treatments approved by the United States Food and Drug Administration (FDA) to treat IC/BPS so far, nevertheless, these treatment show their efficacy only on limited number of patients and can cause side effects (Hanno, 1997; Augé et al., 2020). In addition, several treatments such as hydrodistension, botulinum toxin injections, neuromodulation, and cyclosporine A instillation are commonly used in treating IC/BPS (Garzon et al., 2020); however, there are no reports on their long-term efficacy (Sand et al., 2008; Garzon et al., 2020).

Prolotherapy is recognized as one of the most common supportive and alternative treatments for tissue repair promotion. In brief, it is an injection treatment consisting of regular doses of non-biologic solutions to reduce pain and/or promote proliferation of soft tissue. It has been widely used as an alternative management strategy in treating chronic musculoskeletal and arthritic pain (Nair, 2011). This therapeutic approach has been used clinically with high satisfaction and excellent results in patients (Rabago et al., 2019). Dextrose is the most common solution used in prolotherapy, which is an ideal proliferant due to its water solubility and part of a normal component of blood chemistry (Hauser et al., 2011). Previous *in vitro* and *in vivo* studies showed that dextrose triggers the production of growth factors to facilitate tissue regeneration as well as the growth of ligaments and tendons, fibroblastic proliferation, and the extracellular matrix and articular cartilage repair (Hauser et al., 2016; Sit et al., 2016). A study revealed that dextrose prolotherapy can suppress the infiltration response of macrophages and facilitate regeneration of muscle satellite cells after contusion injury of muscle (Tsai et al., 2018). In addition, hypertonic dextrose was demonstrated to be beneficial for stimulating direct intracellular expression of growth factors in tenocytes and fibroblasts (Vora et al., 2012). This may be one of the mechanisms that hasten chronic wound healing on prolotherapy.

In this study, we aimed to investigate the effects of dextrose prolotherapy in a rat model of IC/BPS and patients with IC/BPS. We used different route of dextrose administration in both studies: intravesical instillation was performed in rat model of IC/BPS and intravesical injection was performed in patients with IC/BPS. Firstly, to initiate rat bladder inflammation, cyclophosphamide (CYP) was used to induce interstitial cystitis in rats (Augé et al., 2020). CYP is an alkylating anti-tumor agent commonly used in the treatment of lymphoma and leukemia. CYP is metabolized by hepatic enzymes, such as cytochrome P450, to produce urinary metabolites (acrolein). The retention of that product in the bladder will cause damage leading to hemorrhagic cystitis and common urinary irritation symptoms such as frequent urination, painful urination, and urgency. Since CYP has the side effects of inducing IC/BPS symptoms, it is usually used in experimental animal models to identify pathogenesis or molecular regulation pathways or to formulate clinical treatment strategies (Birder and Andersson, 2018). Further, as dextrose can promote wound healing by secretion of ECM, we hypothesized that dextrose prolotherapy may support urothelium repair and relieve pain in IC/BPS. According to the findings of the IC/BPS animal study, we thus performed dextrose treatment for clinical use. Hereby, we present a novel clinical strategy to promote the ECM synthesis in damaged urothelium in IC/BPS.

## Materials and Methods

### Animal Model of Interstitial Cystitis/Bladder Pain Syndrome

**FIGURE 1:**
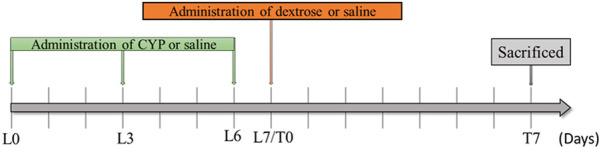
Experimental design of dextrose prolotherapy in IC rat model. (L0 = Lesion Day 0; L3 = Lesion Day 3; L6 = Lesion Day 6; L7/T0 = Lesion Day 7/Treatment Day 0; T7 = Treatment Day 7; CYP = cyclophosphamide).

A total of 32 eight-week-old Sprague Dawley female rats weighing ∼220–250 g (Lesco Biological Co., Ltd., Taiwan, R.O.C) were used in this study. All animal experiment procedures were approved by the Institutional Animal Care and Use Committee (IACUC-20-276) at the National Defense Medical Center (Taipei, Taiwan, R.O.C). The rats were randomly assigned to the normal control group (*n* = 8), cyclophosphamide-induced interstitial cystitis group (CYP) (*n* = 8), cyclophosphamide-induced interstitial cystitis treated with physiological saline prolotherapy group (CYP + Saline) (*n* = 8), and cyclophosphamide-induced interstitial cystitis treated with dextrose prolotherapy group (CYP + Dextrose) (n = 8). In the normal control group, 0.9% physiological saline was injected intraperitoneally, while in the CYP group, CYP (Sigma-Aldrich, St. Louis, MO, United States) at a dose of 75 mg/kg once every 3 days using three consecutive injections (Lesion Day 0, L0; Lesion Day 3, L3; Lesion Day 6, L6) was administered intraperitoneally to induce interstitial cystitis in rats (Augé et al., 2020). On the seventh day, intravesical instillation of the 10% dextrose solution was performed (Lesion Day 7/Treatment Day 0, L7/T0), and the rats were then sacrificed for specimen sampling and examination on the 14th day (Treatment Day 7, T7) (Figure 1).

### Dextrose Prolotherapy in IC Rat Model

Briefly, the rats were fasted for 12 h prior to bladder perfusion and anesthetized with isoflurane (Baxter, USA) by inhalation with a respirator. After the rat was deeply anesthetized, its lower abdomen and the surrounding area of the urinary tract opening were shaved. The urethral opening area was then disinfected with 10% povidone-iodine. Further, a polyethylene catheter (Polyethylene Tubing 50, PE50, BD, USA) was subsequently clamped with sterile instruments for urethral intubation. After the catheter was installed, the rat’s bladder was lightly pressed by hand to drain the urine through the polyethylene catheter. A 1-ml syringe was used to aspirate 500 μl of 10% dextrose solution. Afterward, the needle was connected to the polyethylene catheter, and the solution was slowly injected into the rat’s bladder. The catheter was then removed, and the dextrose solution was retained in the bladder for 30 min. Furthermore, the breathing gas was removed to allow the rat to wake up naturally, and the rat’s condition was then observed after the operation.

### Urodynamic Test

Urodynamic test was performed as described previously (Meng et al., 2011). Amino potassium (Urethane, Ferak, Germany) was intraperitoneally administered to rats with a dose of 1–1.5 mg/kg, and the effect was achieved after 15–20 min. The heating lamp was adjusted throughout the process to maintain the ambient temperature at 25°C–28°C to prevent the rats from losing the temperature. The detection program (LabChart7-v6, ADInstruments, New Zealand) was started to perform for two-point calibration, and a 30-cm-high water column and a large air pressure column was used as the calibration standard. Further, the skin in the lower abdomen was opened, and the muscle was carefully torn open with sharp tweezers to avoid damaging the bladder. The bladder was then removed and gently secured at the base with a single knot of no. 5 black silk. A 26G needle was used to puncture the muscle layer at the top of the bladder to create a small wound, from which a small incision was made in the uroepithelium. A polyethylene catheter was inserted into the wound and tied tightly with a single knot of no. 5 black silk thread (Pearsalls, Taunton, United Kingdom). Afterward, the bladder was placed back into the abdominal cavity, and the wound was covered with a 0.9% saline-soaked cotton ball after simple suturing of the epithelium. Then, the other end of the polyethylene catheter was connected to the pressure sensor. The injection motor was further turned on, and the water supply rate was adjusted to 3 ml of water per hour. Finally, the contraction pressure, volume, and compliance of the bladder were recorded using a program. A stable cystometrogram was selected to calculate bladder pressure and voiding volume changes, threshold pressure, maximum bladder pressure, and inter-contraction interval.

### Hematoxylin and Eosin Staining

The rat’s bladder samples were analyzed using hematoxylin and eosin stain (ScyTeK Laboratories, Logan, UT, United States) according to the manufacturer’s instructions. Firstly, xylene and alcohol were used to remove the paraffin wax on the samples, and then the samples were rinsed with distilled water. Afterwards, hematoxylin stain was dropped on the tissue slides and reacted for 5 min. After the reaction was completed, the slides were washed twice using distilled water and then immersed in alcohol to remove the remaining water. Eosin stain was further dropped on the tissue slides. After 2 min of reaction, the slides were rinsed using anhydrous alcohol three times, and finally, sealed with sealing glue and observed using an inverted microscope (BX53, Olympus, Tokyo, Japan). In this result, the bladder wall thickness was measured using the ImageJ software.

### Immunofluorescence Staining

Firstly, the bladder tissues were fixed in 4% paraformaldehyde. After the fixation was completed, the dehydration was performed in the sucrose gradient. The tissue samples were embedded in an optimal cutting temperature compound (OCT, Thermo Fisher Scientific, Waltham, MA, United States) and sliced transversely with a cryostat (10 μ each). After the tissue section was washed with 0.1 M phosphate-buffered saline (PBS, Sigma-Aldrich, St. Louis, MO, United States), the samples were covered with a blocking solution for 30 min at 27°C. Afterward, the samples were incubated with primary antibodies, including cell surface antigen 44 (CD44) and receptor for hyaluronic acid-mediated motility (RHAMM) (1:500 dilution, Santa Cruz Laboratories, Dallas, TX, United States), at 4°C for overnight and then were washed with 0.1 M PBS the next day. Further, the secondary fluorescent antibodies were added to the samples at room temperature for 60 min. After the reaction, the samples were washed with 0.1 M PBS. A 4′,6-diamidino-2-phenylindole (DAPI, Thermo Fisher Scientific, Waltham, MA, United States) was added to the samples at room temperature for 10 min, followed by washing the samples with 0.1 M PBS and drying them in the darkroom at room temperature. Finally, the samples were covered and mounted within a glass slide, and furthermore, they were observed under a fluorescent microscope (Axio Lab.A1, Carl Zeiss AG, Oberkochen, Germany) embedded with a camera (Zeiss AxioCam ICm1, Carl Zeiss AG, Oberkochen, Germany).

### RNA Isolation, Reverse Transcription, and Quantitative RT-PCR

RNA was extracted from rat bladder tissue using TRIzol reagent (Life Technologies, Grand Island, NY, United States) and quantified using an ultra-micro spectrophotometer (NanoDrop 2000, Thermo Fisher Scientific, Waltham, MA, United States). A 20-µl solution containing 3 µg of RNA was reverse transcribed using the Reverse Transcription Kit (QIAGEN, Germany). A 50 ng of cDNA per well was then mixed with a fixed ratio of fluorescent dye (SYBR Green qPCR Mastermix, QIAGEN, Germany) and RNase-free water. Further, the 20 µl of the mixed solution was added to a 96-well plate, and CD44, hyaluronan synthase 1 (HAS1), hyaluronan synthase 2 (HAS2), hyaluronan synthase 1 (HAS3), and RHAMM gene expressions were assessed with real-time PCR (Applied Biosystems 7500 Real-Time PCR System, Applied Biosystems, USA). The results were normalized to GAPDH levels according to the –∆∆Ct method (where Ct is the threshold cycle). LightCycler 480 v1.5.0 (Roche Applied Science, Penzberg, Germany) was used to analyze PCR kinetics and mRNA using the standard curve method.

### Patient Enrollment

This study received ethical approval from the Institutional Review Board (2-108-05-062) and the General Clinical Research Center of the Tri-Service General Hospital, Taipei, Taiwan. The study was registered with clinicaltrials.gov (NCT04821882). We consecutively recruited 32 patients with a clinical diagnosis of IC/BPS. The inclusion criteria were: 1) the subject is aged over 20 years old; 2) the subject was diagnosed as IC/BPS; 3) the subject with lower urinary tract symptoms, such as frequent urination, urgent urination, or bladder pain; 4) treated with intravesical instillations of hyaluronic acid (HA) and/or Botox for more than 6 months. The exclusion criteria were: 1) the subject is aged under 20 years old; 2) pregnant women; 3) the subject with congenital disorders of the urinary tract; 4) the subject with a urinary tract infection, Hunner lesions, tumor lesions, any pathological change lesions, or stones. Three subjects of 32 cases were further excluded from the study due to personal reasons. Hence, a total of 29 patients (aged 40–65 years) with IC/BPS were enrolled in this study from May 2019 to October 2020. All patients were treated with intravesical instillations of hyaluronic acid (HA) and/or Botox for more than 6 months; however, the treatment efficacy was poor, and the patients’ condition remained unchanged. After adequate treatment for IC/BPS in accordance with American Urological Association (AUA) guidelines, these patients still experienced sustained persistent pain, frequency, and urgency of urination. All patients were informed about the study’s rationale and operative procedures. Informed consent was obtained from each patient for this off-label use of intravesical injection with dextrose.

### Patient Intervention

Eligible patients were confirmed for treatment and requested to complete a 3-day voiding diary before treatment. Patients received intravesical injections of 10% dextrose under intravenous general anesthesia in the operating room: 2 ml of 50% Dextrose (Vitagen injection, Taiwan Biotech Co., Ltd., Taipei, Taiwan) was diluted with 10 ml of normal saline and delivered via 11 suburothelial injections (1 ml each site). The injection needle was inserted into the urothelium at the trigone (one site), posterior (six sites), and lateral walls (four sites) of the bladder. Dextrose was injected using a 23-gauge needle and a rigid cystoscopic injection (22-Fr; Richard Wolf, Knittlingen, Germany). We did not provide post-injection analgesics to any of the patients. Patients were observed at a post-anesthesia care unit for half an hour. After self-voiding, they were allowed to go home. Treatment was repeated based on patients’ condition with a maximum of five planned therapy cycles up to 12 weeks. All patients were followed up and assessed at baseline, 2, 4, 8, and 12 weeks after the treatment.

### Patient Outcome Assessment and Follow-Up

Patients were asked to provide a 3-day voiding diary for frequency of daytime and nighttime episodes of urgency, pain, and intake-output records. The overall efficacy outcome of the treatment was measured using several questionnaires including Global Response Assessment (GRA), International Prostate Symptom Score (IPSS-TOTAL), IPSS-voiding (IPSS-V), IPSS-storage (IPSS-S), Visual Analog Scale (VAS), Interstitial Cystitis Symptom Index (ICSI), Interstitial Cystitis Problem Index (ICPI), 5-item Brief Symptom Rating Scale (BSRS-5), and 5-item Female Sexual Function Index (FSFI-5). In GRA questionnaire (Hauser et al., 2011), patients were requested to rate overall pelvic symptoms on seven response categories: 1) markedly worse (−3), 2) moderately worse (−2), 3) slightly worse (−1), 4) no change (0), 5) slightly improved (+1), 6) moderately improved (+2), and 7) markedly improved (+3). Of note, moderately and markedly improved results after treatment (≥2) were considered a successful treatment outcome. The severity of voiding symptoms was evaluated using IPSS-TOTAL, IPSS-V, and IPSS-S sub-scores. Pain intensities were scored via patient self-reported assessments using a 10-point VAS system. IC/BPS symptoms were assessed by the O’Leary-Sant symptom score (OSS) including ICSI and ICPI (Lubeck et al., 2001). The eight questions of the OSS (ICSI/ICPI) score were used to assess pain and voiding symptoms in these patients. The maximal index score of 36 represents the severe symptom and problem, while the lowest index score of 0 reflects the slightest symptom and problem. The quality of life (QoL) of patients was evaluated using BSRS-5 questionnaire (Chen et al., 2005). In addition, the sexual dysfunction in female patients was assessed using FSFI-5 questionnaire (Chung et al., 2019).

Urine samples were collected and stored at 4°C until analysis. Samples were analyzed for the presence of four analytes: 1) epidermal growth factor (EGF), 2) hepatocyte growth factor (HGF), 3) placental growth factor-1 (PIGF-1), and 4) vascular endothelial growth factor-D (VEGF-D). Their concentrations were measured using a Growth Factor 11-Plex Human ProcartaPlex™ Panel assay kit with magnetic beads (EPX110-12170-901, Thermo Fisher Scientific, Waltham, MA, United States) and examined with an automated immunoassay analyzer (Luminex™ 100 IS System, Luminex, TX, United States) and the accompanying ProcartaPlex Analyst Software 1.0 (Thermo Fisher Scientific, Waltham, MA, United States) according to the manufacturer’s instructions.

For the cytokine assay, the levels of serum cytokines [interleukin-6 (IL-6), IL-10, IL-12p70, IL-13, IL-17F, and IL-27] in blood samples were detected using a magnetic bead-based multiplex immunoassay MILLIPLEX MAP Human Th17 Magnetic Bead Panel (Merck KGaA, Darmstadt, Germany) according to the manufacturer’s protocol. The MAGPIX® System reader was used to acquire the data of cytokines, which was reported as concentrations (pg/ml) using the Belysa™ Immunoassay Curve Fitting Software (Merck KGaA, Darmstadt, Germany).

The information on the functional bladder, the score of questionnaires (GRA, IPSS-TOTAL, IPSS-V, IPSS-S, VAS, ICSI, ICPI, BSRS5, and FSFI5), and urine samples for Luminex assay of growth factors were collected at every follow-up, while blood samples for cytokine assay were collected at 12 weeks after the treatment period. In addition, a total of 19 healthy subjects was recruited to be served as control comparison for Luminex assay of growth factors and cytokine analyses.

### Statistical Analysis

The statistical analysis was performed using SPSS, version 18.0 (SPSS Inc., IL, United States). Data were expressed as mean values ± standard deviations. The analyses of the urinary voiding symptoms, the score of questionnaires (GRA, IPSS-TOTAL, IPSS-V, IPSS-S, VAS, ICSI, ICPI, BSRS5, and FSFI5), and Luminex assay result of growth factors were evaluated by comparing pre- and post-treatment using paired t-test. Mann-Whitney analysis and Bonferroni correction were applied for multiple comparisons between the groups. Correlation analysis and correlation coefficient were performed for comparing the VAS questionnaire and growth factor relation. A *p*-value of ≤0.05 was considered significant.

## Results

### Urodynamic Test

**FIGURE 2:**
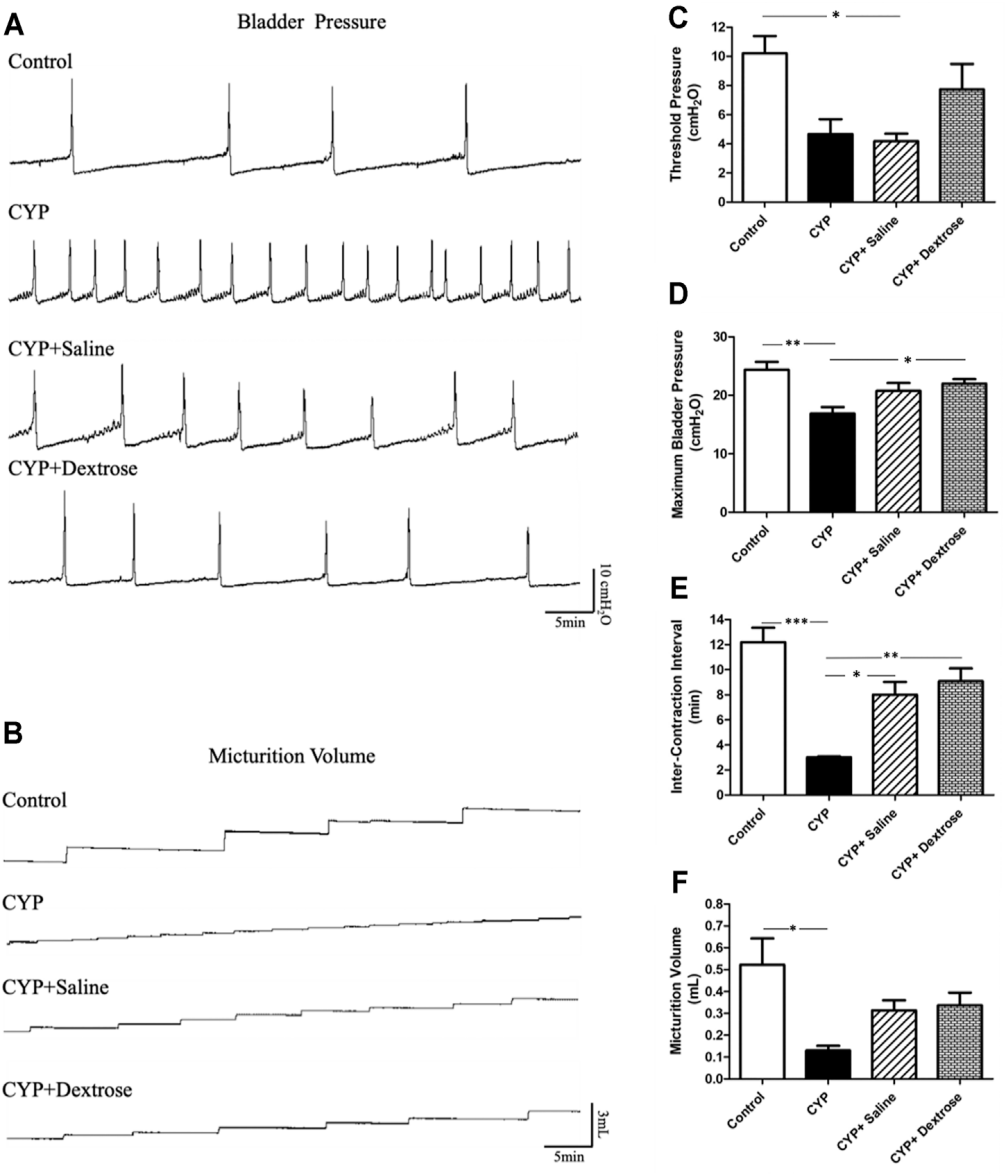
Cystometric voiding parameters of rats with interstitial cystitis in response to treatment. **(A)** Cystometrogram recordings of bladder pressure; **(B)** cystometrogram recordings of micturition volume; **(C)** quantitative of threshold pressure; **(D)** quantitative of maximum bladder pressure; **(E)** quantitative of inter-contraction interval; **(F)** quantitative of micturition volume. (CYP = cyclophosphamide; *p ≤ 0.05; **p ≤ 0.01; ***p ≤ 0.001).

In this experiment, the bladder contraction pressure, volume, and the number of urinations were recorded when the bladder was infused to the natural urination and contraction. Cystometrogram recordings of bladder pressure displayed a significant increase in the frequency of involuntary bladder urination and contraction in all experimental groups compared to the normal control group (Figure 2A). Further, CYP and CYP-saline groups showed significantly reduced maximum bladder pressure (*p* ≤ 0.01) and level of threshold pressure (*p* ≤ 0.05), respectively, compared to the normal control group, while the CYP-dextrose group showed no significant difference (Figures 2C,D). Regarding inter-contraction interval and micturition volume, the values in CYP group was also significantly lower (*p* ≤ 0.001, *p* ≤ 0.05, respectively) when compared to the normal control group, while there was no significant difference in the CYP-saline and CYP-dextrose groups (Figures 2E,F). Moreover, we found that maximum bladder pressure and inter-contraction interval, implying the interval between urination, were significantly increased after the prolotherapy (CYP-saline (*p* ≤ 0.05) and CYP-dextrose groups (*p* ≤ 0.05 and *p* ≤ 0.01), respectively) compared to the CYP group, and the highest value was observed in the CYP-dextrose group (Figures 2D,E). In addition, the cystometrogram recordings of micturition volume in the CYP-dextrose group was relatively similar compared to the normal control group (Figure 2B). Overall, these results revealed that the CYP-induced interstitial cystitis in rats changed the critical threshold and the maximum pressure of urination, increasing the frequency of urination, and decreasing the volume of the bladder. However, the administration of dextrose into the bladder could improve the instability of the bladder and reduce frequent urination.

### Histological Analysis

**FIGURE 3:**
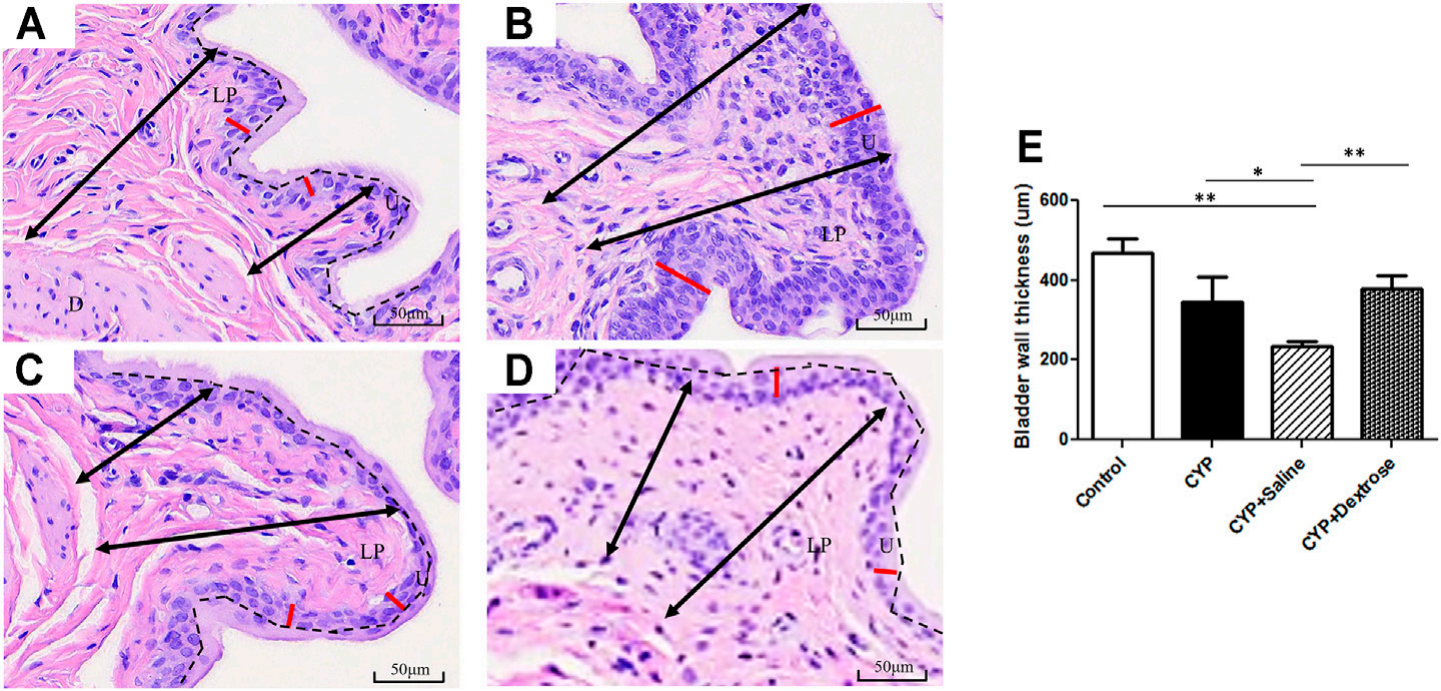
Hematoxylin and eosin staining of bladder tissues in interstitial cystitis rat model. **(A)** Normal control group; **(B)** CYP group; **(C)** CYP-saline group; **(D)** CYP-dextrose group; **(E)** Quantitative analysis of the bladder wall thickness. (D = detrusor; LP = lamina propria; U = urothelium; black arrow = the range of bladder wall; red line = the urothelium; black dash = the separation of glycosaminoglycan layer and urothelium; *p ≤ 0.05; **p ≤ 0.01).

**FIGURE 4:**
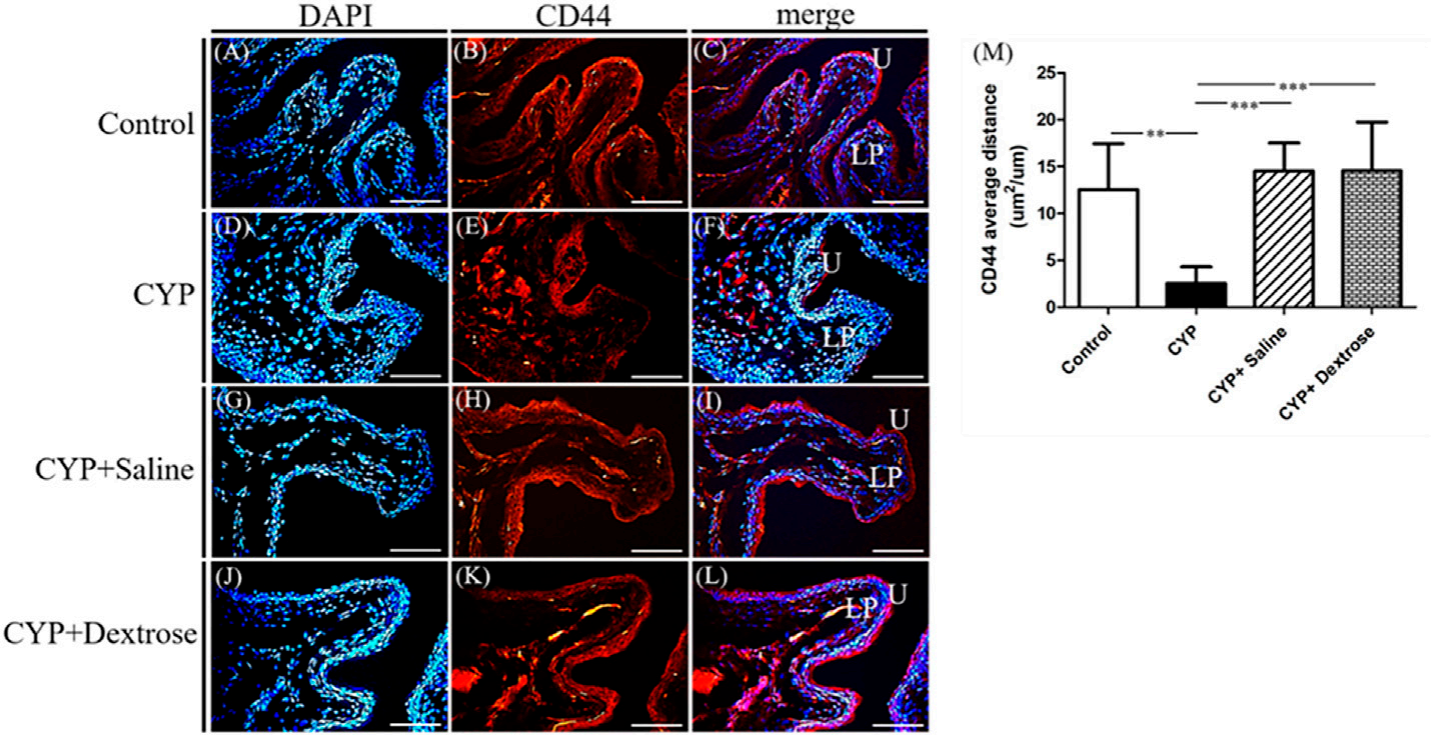
Immunofluorescence staining and quantitative analysis of cell surface antigen 44 (CD44) marker of bladder tissues in interstitial cystitis rat model. **(A)** Control, DAPI; **(B)** control, CD44; **(C)** control, merge DAPI + CD44; **(D)** CYP, DAPI; **(E)** CYP, CD44; **(F)** CYP, merge DAPI + CD44; **(G)** CYP + saline, DAPI; **(H)** CYP + saline, CD44; **(I)** CYP + saline, merge DAPI + CD44; **(J)** CYP + dextrose, DAPI; **(K)** CYP + dextrose, CD44; **(L)** CYP + dextrose, merge DAPI + CD44; **(M)** quantitative analysis of CD44. (CYP = cyclophosphamide; DAPI = 4′,6-diamidino-2-phenylindole; U = urothelium; LP = lamina propria; scale bar = 50 μm; *p ≤ 0.05; **p ≤ 0.01; ***p ≤ 0.001).

**FIGURE 5:**
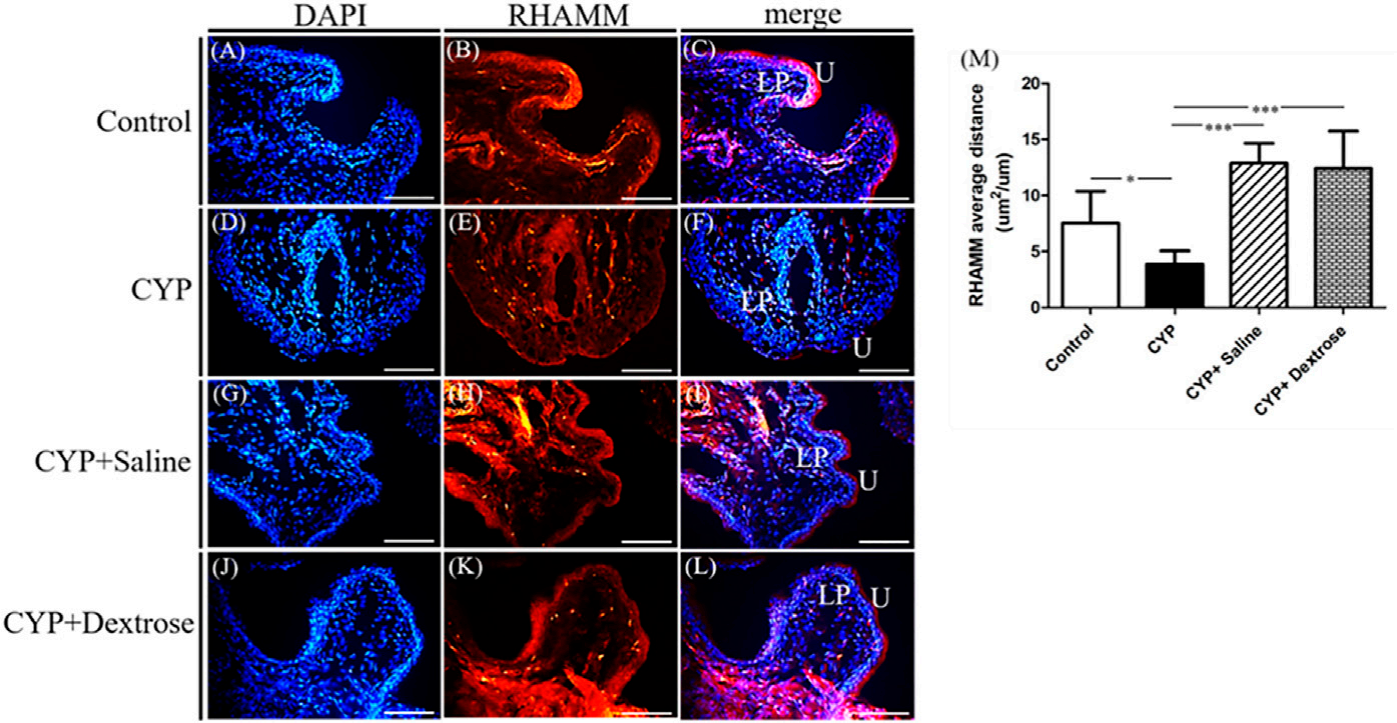
Immunofluorescence staining and quantitative analysis of receptor for hyaluronic acid-mediated motility (RHAMM) of bladder tissues in interstitial cystitis rat model. **(A)** Control, DAPI; **(B)** control, RHAMM; **(C)** control, merge DAPI + RHAMM; **(D)** CYP, DAPI; **(E)** CYP, RHAMM; **(F)** CYP, merge DAPI + RHAMM; **(G)** CYP + saline, DAPI; **(H)** CYP + saline, RHAMM; **(I)** CYP+saline, merge DAPI + RHAMM; **(J)** CYP + dextrose, DAPI; **(K)** CYP + dextrose, RHAMM; **(L)** CYP + dextrose, merge DAPI + RHAMM; **(M)** quantitative analysis of RHAMM. (CYP = cyclophosphamide; DAPI = 4′,6-diamidino-2-phenylindole; U = urothelium; LP = lamina propria; scale bar = 50μm; *p ≤ 0.05; **p ≤ 0.01; ***p ≤ 0.001).

For H&E staining results, we observed that the urinary tract of the CYP group was less dense and discontinuous with abundant darker nuclei and more loosely arranged cells in the basement membrane with disrupted glycosaminoglycan layer when compared to the normal control group (Figures 3A,B). In contrast, in the CYP-saline and CYP-dextrose groups, the urothelium was more similar to the normal control group (Figures 3C,D). The bladder wall thickness, which is the distance from bladder epithelial cell to the bladder muscle, in the CYP group was slightly thicker than the control group, however, no significance was found (Figure 3E). In the CYP-saline group, it was significantly thinner than the control group (*p* ≤ 0.01). Moreover, the bladder wall thickness in the CYP-dextrose was significantly thicker when compared to the CYP-saline group (*p* ≤ 0.01), even though it had no significance when compared to the control and CYP groups. Nevertheless, in terms of the epithelium and urothelium structures, glycosaminoglycan layer, and bladder wall thickness, the results demonstrated that the CYP-dextrose group displayed a better tissue repair. Further, immunofluorescence staining was performed to examine the distribution and gene expression of CD44 and RHAMM during the regeneration phase of the bladder tissue after the prolotherapy. For quantitative immunofluorescent staining results, the red fluorescence stain referred to as positive for HA receptors, by dividing hyaluronic acid-mediated motor receptors with the urothelial length (μm^2^/μm) outside the urothelium. The distribution of CD44 expression and RHAMM receptors was found in both the upper layer of the urothelium and the lamina propria of the bladder (Figures 4C,F,I,L, 5C,F,I,L). The intensity of the CD44 fluorescence both in the CYP-saline and CYP-dextrose groups was significantly increased (*p* ≤ 0.001, respectively) compared to that in the CYP group, and CD44 expression in the CYP group was significantly decreased (*p* ≤ 0.01) compared to the normal control group (Figure 4M). Further, the fluorescence intensity of RHAMM receptors in the epithelial layer of the bladder in the CYP group was significantly decreased (*p* ≤ 0.05) compared to the normal control group (Figure 5M), while in contrast, both CYP-saline and CYP-dextrose groups demonstrated a similar tendency to that of the normal control group and the intensity was significantly increased (*p* ≤ 0.01, respectively) compared to that in the CYP group (Figure 5M). These data indicate that dextrose prolotherapy could improve the disrupted urothelial barrier and increase the thickness of the bladder wall, by restoring it to two to three layers.

### Extracellular Matrix-Related Gene Expression

**FIGURE 6:**
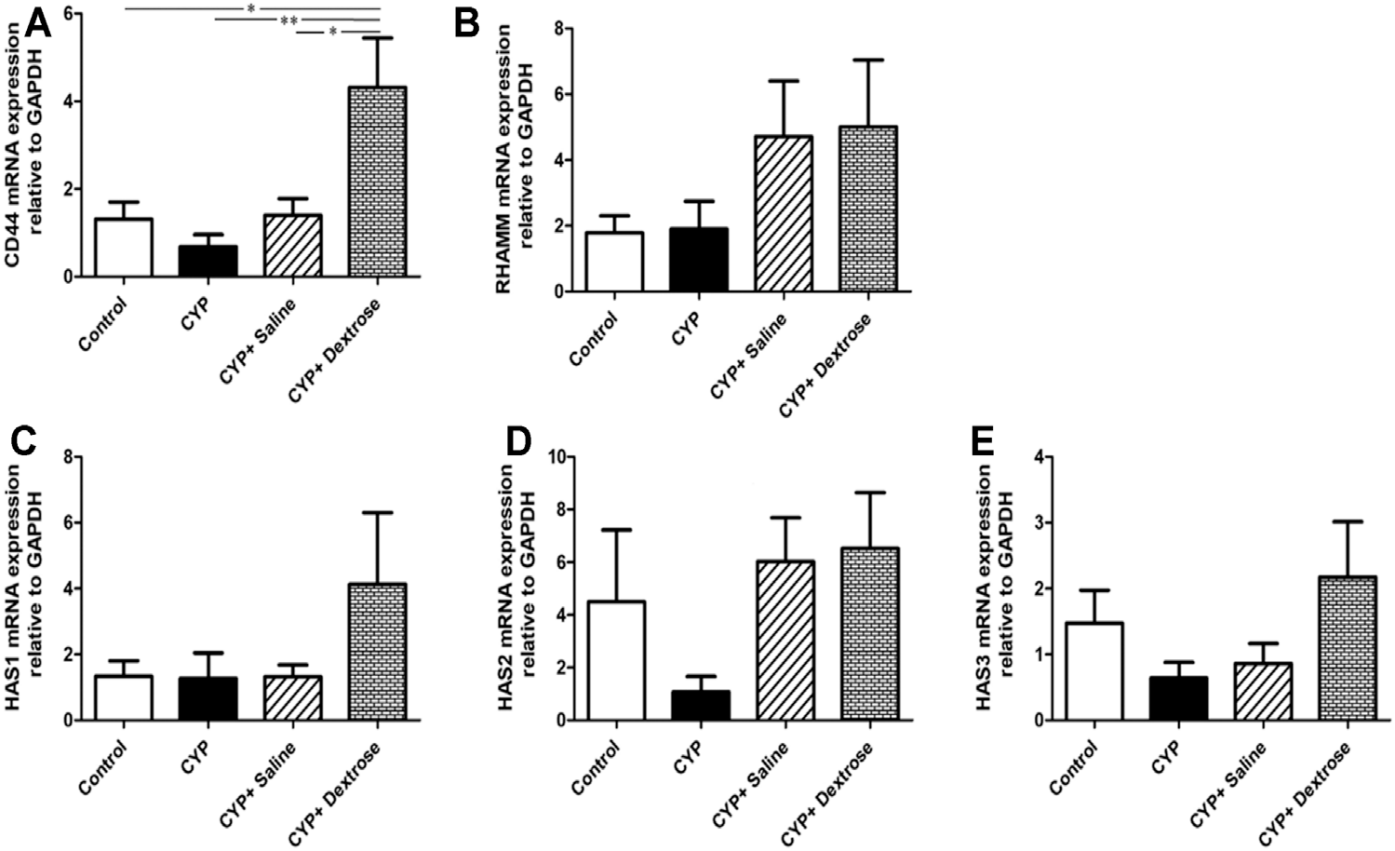
mRNA expression of bladder tissues in interstitial cystitis rat model. **(A)** Cell surface antigen 44 (CD44); **(B)** receptor for hyaluronic acid-mediated motility (RHAMM); **(C)** hyaluronic acid synthesis 1 (HAS1); **(D)** hyaluronic acid synthesis 2 (HAS2); **(E)** hyaluronic acid synthesis 3 (HAS3). (*p ≤ 0.05; **p ≤ 0.01; ***p ≤ 0.001).

HA is one of the major components of the ECM, and it plays an important role in maintaining the structural integrity and function of the urinary bladder epithelium and tissue repair. Therefore, we analyzed the genetic alterations affecting hyaluronic acid, including hyaluronic acid receptors (CD44 and RHAMM) and hyaluronic acid synthases (HAS1, HAS2, and HAS3). The results demonstrated that the expression of CD44 in the CYP-dextrose group was significantly highest among all groups with *p* ≤ 0.05, *p* ≤ 0.001, and *p* ≤ 0.05, respectively (Figure 6A). Further, the expression of RHAMM, HAS1, HAS2, and HAS3 also showed a similar tendency, however, no significant differences were found (Figures 6B–E).

### Patients Characteristics, Cumulative Success Rate, and the Effect of Dextrose Prolotherapy in Urinary Voiding Symptoms

**FIGURE 7:**
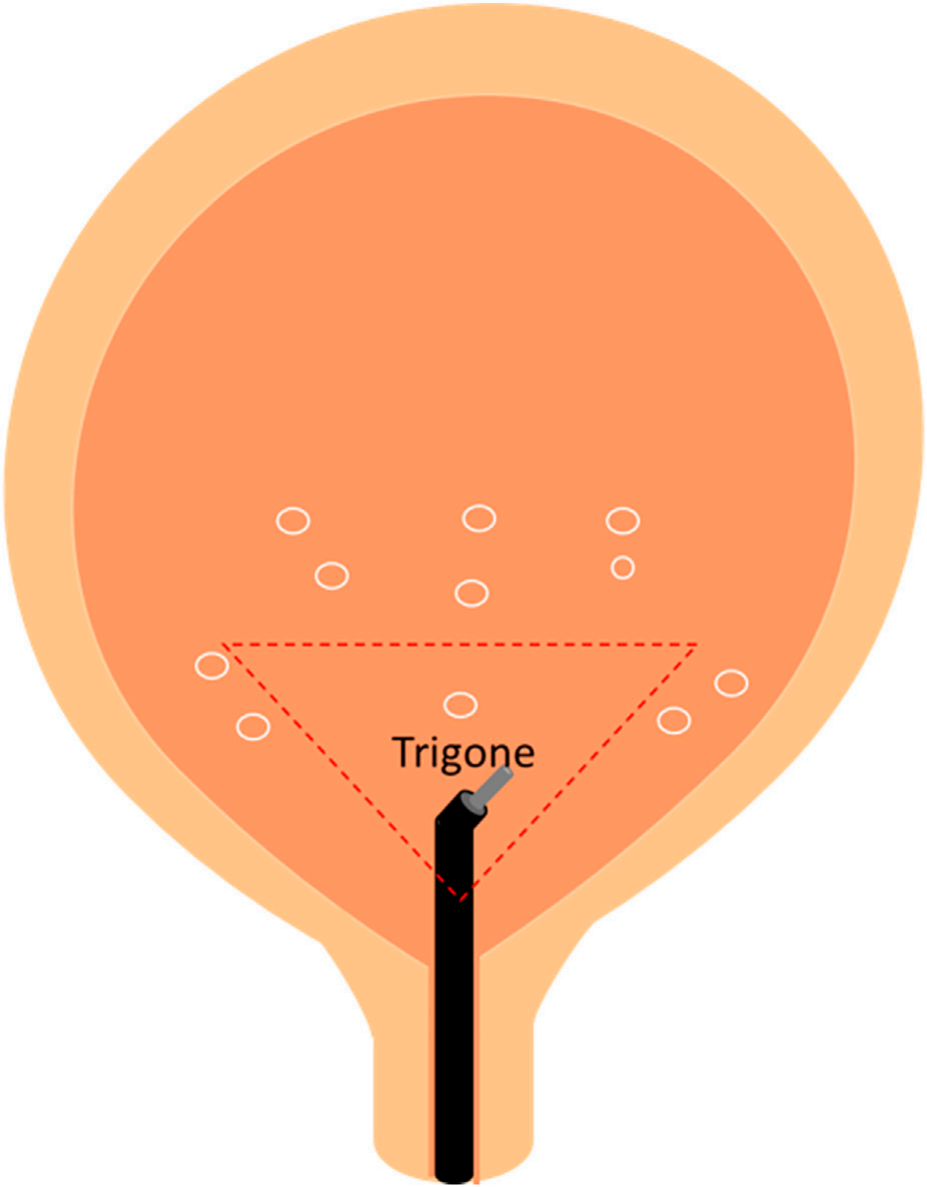
Location of dextrose injection sites.

**FIGURE 8:**
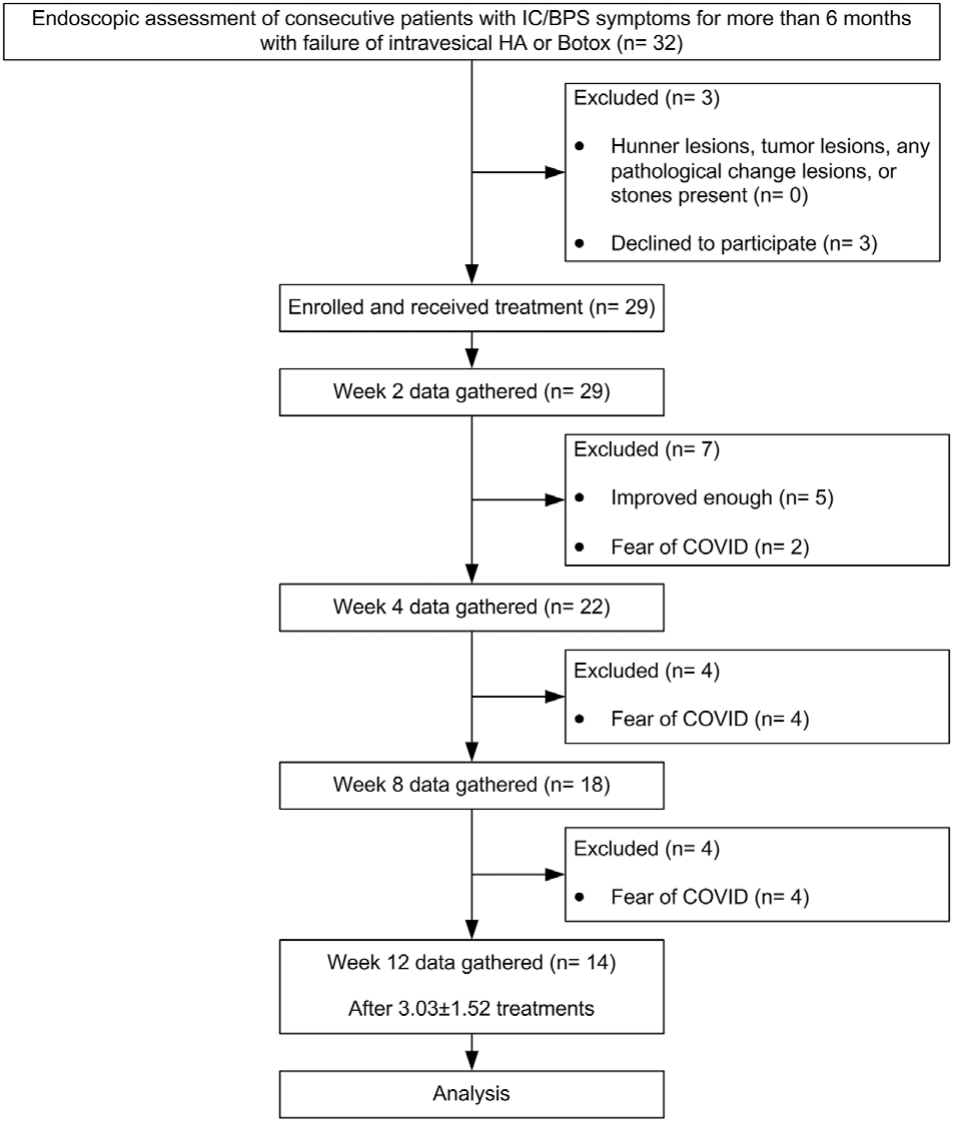
Flowchart of the study.

**FIGURE 9:**
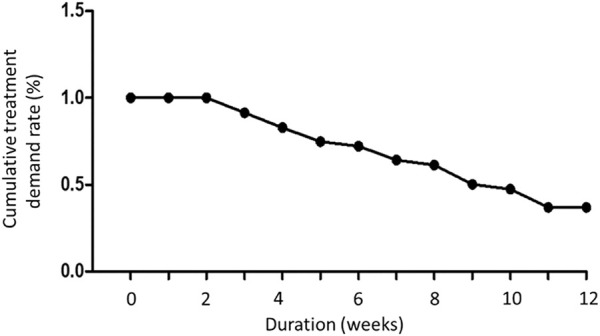
The cumulative treatment demand rate of dextrose prolotherapy during 12 weeks of treatment period.

A total of 29 patients were enrolled in this study. Patient background information are displayed in Table 1. Until the end of the treatment period, only 14 patients completed the entire follow-up assessments, and their data were analyzed. The location of dextrose injection sites and the flowchart of the study are demonstrated in Figures 7, 8, respectively. The planned maximum of dextrose prolotherapy was five times; however, it varied for each patient. Most patients showed improvement before the completion of planned treatment and thus abandoned the further follow-up, therefore, they were excluded from the study. The cumulative treatment demand rate after the first intravesical injection with dextrose is shown in Figure 9; it implies that the need for dextrose prolotherapy decreased as time progressed. The cumulative treatment demand rate was calculated as the number of patients who continued treatment during the week divided by the number of patients treated in the previous week and the demand rate of the previous week (Vittinghoff et al., 2005). Major storage symptoms (i.e., urinary frequency, urgency, nocturia, and maximum bladder capacity) were recorded and are listed in Table 2. The results demonstrate that the frequency of nocturia significantly decreased after 12 weeks of treatment, whereas other symptoms had no remarkable change.

**TABLE 1:** Patient background characteristics.

Patient characteristics	Value
Sex, n	
Male	2
Female	27
Ages (year, mean)	55.17 ± 12.15
Previous treatment	Hyaluronic acid
	Botox
	IC/BPS treatment (AUA guidelines)
Duration (month)	≥ 6

**TABLE 2:** Urinary voiding symptom score of patients with interstitial cystitis/bladder pain syndrome according to bladder diary during 12 weeks of dextrose prolotherapy.

	W0	W2	W4	W8	W12
Urinary frequency	13.44 ± 2.06	12.33 ± 1.53	11.11 ± 0.82	9.78 ± 0.91	12.43 ± 1.25
Urinary urgency	6.13 ± 2.96	3.00 ± 2.45	4.14 ± 1.78	3.43 ± 1.79	9.00 ± 1.47
Nocturia	2.56 ± 0.56	3.00 ± 0.41	2.78 ± 0.49	2.22 ± 0.36	2.00 ± 0.31*
Maximum bladder capacity	321.37 ± 16.99	350.03 ± 28.22*	372.85 ± 18.32	384.28 ± 21.21*	393.75 ± 19.90

*p ≤ 0.05.

### Questionnaires Assessment of Dextrose Prolotherapy Effect

Table 3 summarizes the results of all questionnaire assessments. When compared with baseline, no significant difference was noted in GRA and FSFI-5 questionnaires at each time point of assessment. However, there was a remarkable decrease in scores of other questionnaires: IPSS-TOTAL, IPSS-S, and VAS after 2 weeks of treatment, IPSS-V, ICSI, and ICPI after 4 weeks of treatment, and BSRS-5 after 8 weeks of treatment. Mean scores of IPSS-TOTAL, IPSS-S, and VAS reduced significantly by 17.57 ± 2.67 (*p* ≤ 0.01), 7.36 ± 1.08 (*p* ≤ 0.01), and 3.46 ± 0.62 (*p* ≤ 0.05) at week 2 after dextrose prolotherapy. On week 4 following dextrose injection, we observed significant improvement in IPSS-V, ICSI, and ICPI as 8.50 ± 1.47 (*p* ≤ 0.01), 9.42 ± 1.32 (*p* ≤ 0.05), and 9.67 ± 1.20 (*p* ≤ 0.01). However, BSRS-5 was most significantly reduced at week 8 following dextrose treatment (6.80 ± 1.28, *p* ≤ 0.05).

**TABLE 3:** Summary of Global Response Assessment (GRA), International Prostate Symptom Score (IPSS-TOTAL), IPSS-voiding (IPSS-V), IPSS-storage (IPSS-S), visual analog scale (VAS), O’Leary-Sant Interstitial Cystitis Symptom Index (ICSI) and Interstitial Cystitis Problem Index (ICPI), 5-item Brief Symptom Rating Scale (BSRS-5), and 5-item Female Sexual Function Index (FSFI-5) questionnaires assessment.

	W0	W2	W4	W8	W12
GRA	1.50 ± 0.31	1.79 ± 0.30	2.08 ± 0.26	1.90 ± 0.31	1.57 ± 0.20
IPSS_TOTAL	24.71 ± 1.48	17.57 ± 2.67**	15.33 ± 2.30***	15.44 ± 2.87**	16.63 ± 2.19**
IPSS_S	10.79 ± 0.71	7.36 ± 1.08**	6.83 ± 0.90**	7.00 ± 1.12**	7.38 ± 1.08*
IPSS_V	13.21 ± 1.10	10.29 ± 1.70	8.50 ± 1.47**	8.44 ± 1.83*	9.25 ± 1.54**
VAS	5.31 ± 0.60	3.46 ± 0.62*	2.62 ± 0.54***	2.60 ± 0.78***	3.22 ± 0.70**
ICSI	12.77 ± 0.74	10.23 ± 0.91	9.42 ± 1.32*	10.40 ± 1.42	9.56 ± 1.08*
ICPI	13.46 ± 0.64	11.23 ± 1.63	9.67 ± 1.20**	10.09 ± 1.31*	9.78 ± 1.13**
BSRS_5	9.46 ± 1.65	7.46 ± 1.44	8.00 ± 1.26	6.80 ± 1.28*	7.13 ± 1.27*
FSFI_5	43.60 ± 6.71	42.90 ± 7.29	39.00 ± 9.59	47.40 ± 12.01	30.67 ± 11.67

*p ≤ 0.05; **p ≤ 0.01; ***p ≤ 0.001.

### Dextrose Prolotherapy Effect on Growth Factors Alteration

**FIGURE 10:**
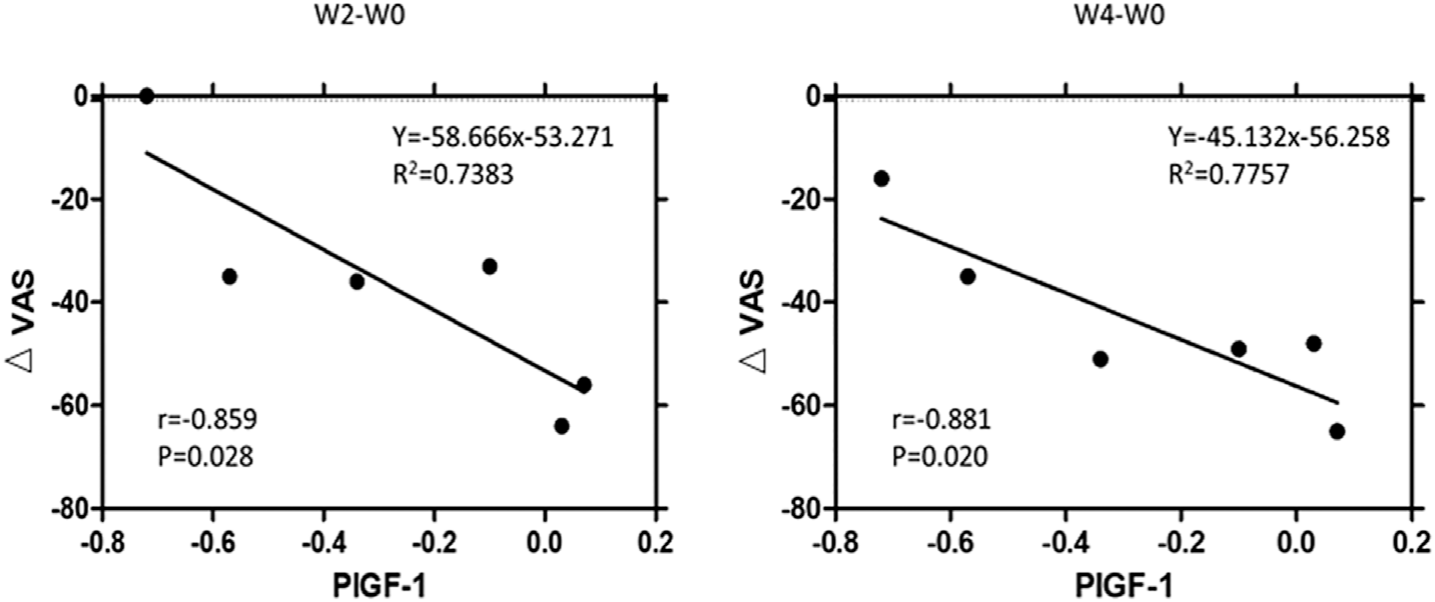
Correlation between the significant decrease of urinary concentration of placental growth factor-1 (∆PIGF-1) and significant decrease of pain intensity (∆VAS; VAS = visual analog scale) at 2-week and 4-week follow-up. R2 = multivariate coefficient of determination.

The alterations of growth factors, including EGF, HGF, placental growth factor-1 (PIGF-1), and vascular endothelial growth factor-D (VEGF-D), in IC/BPS patients, are shown in Table 4 to display the differences before and after dextrose prolotherapy along with their comparison with healthy controls at 2, 4, 8, and 12 weeks of follow-up. Overall, dextrose prolotherapy significantly enhanced the level of EGF and, in contrast, reduced the level of HGF, PIGF-1, and VEGF-D after several weeks following treatment. When compared to healthy controls, the levels of most growth factors, except HGF, had no significant difference in IC/BPS patients at each time point of dextrose prolotherapy. On week 8 following dextrose injection, there was a significant increase of 27.80 ± 4.08 (*p* ≤ 0.01) in HGF. Furthermore, at 2-week and 4-week follow-up, a strong correlation was found between the decrease in PIGF-1 concentration and decrease in pain intensity on the VAS questionnaire (Figure 10).

**TABLE 4:** Urinary concentration of epidermal growth factor (EGF), hepatocyte growth factor (HGF), placental growth factor-1 (PIGF-1), and vascular endothelial growth factor-D (VEGF-D) in IC/BPS patients before and after dextrose prolotherapy and its comparison between IC/BPS patients and healthy patients, respectively.

	Healthy patients	W0	W2	W4	W8	W12
EGF	51,330.97 ± 41392.25	4,889.17 ± 336.83	10,551.15 ± 1162.70***	72436.88 ± 58769.26	23531.56 ± 7774.70	10599.93 ± 3061.02
HGF	15.84 ± 1.36	32.19 ± 4.88	20.91 ± 7.22	15.37 ± 1.31**	27.80 ± 4.08^††^	16.26 ± 5.52
PIGF_1	0.54 ± 0.04	0.75 ± 0.06	0.52 ± 0.09	0.53 ± 0.04**	0.62 ± 0.07	0.42 ± 0.06*
VEGF_D	0.49 ± 0.05	1.05 ± 0.02	0.54 ± 0.14**	0.62 ± 0.08***	0.81 ± 0.16	0.49 ± 0.13*

Unit = pg/ml; *p ≤ 0.05; **p ≤ 0.01; ***p ≤ 0.001; ^††^p ≤ 0.01; * = comparison between IC/BPS patients before and after dextrose prolotherapy; ^†^ = comparison between IC/BPS patients and healthy patients.

### Dextrose Prolotherapy Effect on T Helper Cells Responses

**FIGURE 11:**
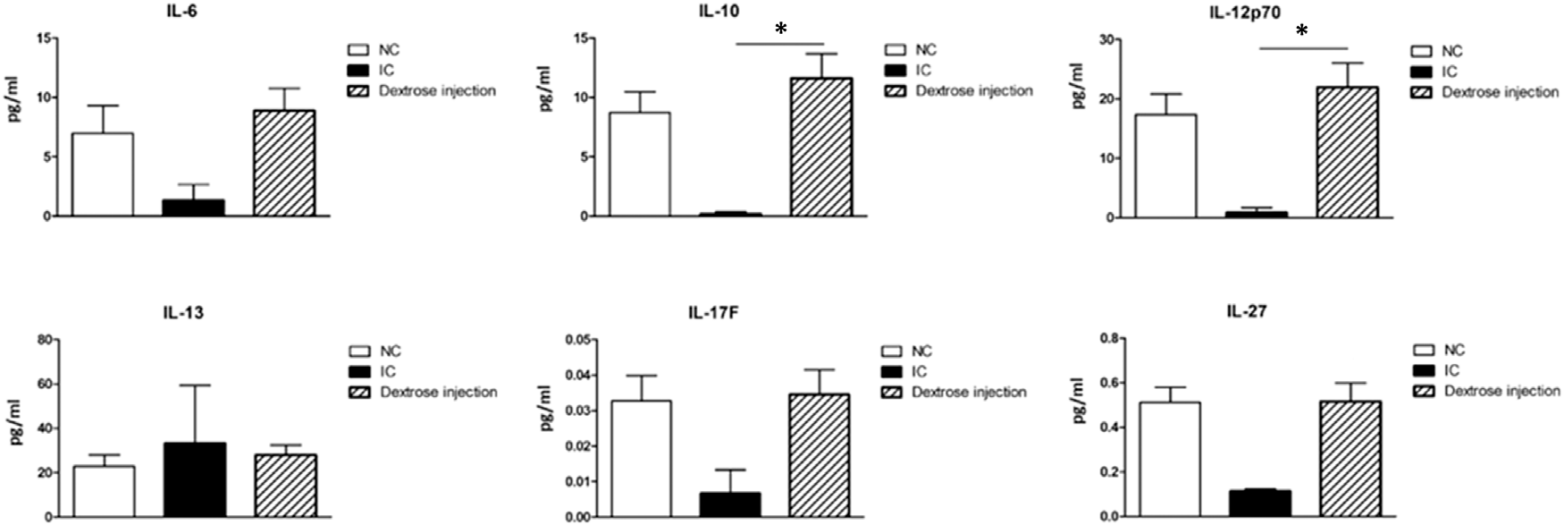
Comparison of serum concentrations of cytokine (IL-6, IL-10, IL-12p70, IL-13, IL-17F, and IL-27) between healthy controls (NC) and IC/BPS patients before and after dextrose prolotherapy. *p ≤ 0.05 was considered significant.

Cells that play a key role in the adaptive immune system assist immune cell function by releasing T cell cytokines. As shown in Figure 11, our results demonstrate that the expressions of Th1 type cytokines IL-12p70 and Treg type cytokine IL-10 were significantly up-regulated after dextrose prolotherapy in IC/BPS patients when compared to before treatment, whereas no significant differences were found for the expressions of Th17 type cytokines IL-6 and IL-17F, Th2 type cytokine IL-13, and IL-27. However, it is also worth noting that there was a similar tendency of these cytokines level in IC/BPS patients after dextrose prolotherapy when compared to healthy controls.

## Discussion

Current treatments of IC/BPS have controversial efficacy, particularly on pain symptoms. Currently, studies with elevated levels have implicated a critical role of HA metabolism in the pathophysiology and resented that the homeostasis between the regeneration of urothelial HA and cell–ECM could modulate the urothelial layer of the bladder regeneration. HA is the major component of the glycosaminoglycan layer of the urothelium in the bladder. HA regulates various cellular activities from migration, proliferation, differentiation, and inflammation to cell-cell interactions and cell–ECM adhesions (Chen and Abatangelo, 1999; Croce et al., 2001; Reitinger and Lepperdinger, 2013). HA is synthesized by the membrane protein hyaluronic acid synthase or HA synthases. In the vertebrates, there are three hyaluronan synthases including HAS1, HAS2, HAS3, and hyaluronic acid receptors (CD44 and RHAMM) among other interactions to regulate cell migration, proliferation, and differentiation (Kaya et al., 1997; Zaman et al., 2005; Mondalek et al., 2015; Lee et al., 2017); these are widely expressed in many cell types in the interstitial tissue. In particular, CD44 primarily acts in the regulation of keratinocyte proliferation in response to extracellular stimuli (Reitinger and Lepperdinger, 2013). In addition, RHAMM as a regulator of CD44–ERK-1/2 fibroblast signalling, is required for mucosal regeneration and to improve urothelial lining defects in cystitis rats (Iavazzo et al., 2007; Ahmad et al., 2008; Cervigni et al., 2008; Lee et al., 2017). Our animal study revealed that after dextrose prolotherapy, the regeneration of the uroepithelium glycosaminoglycan layer and the gene expression of the HA and HAS receptors were increased in the CYP-induced rats (Figures 3, 4, 5). The HA was initially synthesized on the inner surface of the cell by HAS and released into the extracellular space. It improved the mobility of urothelial cells and smooth muscle cells and increased the bladder epithelium glycosaminoglycans. Notably, the expression of CD44 between the CYP-saline and CYP-dextrose groups were significantly different (Figure 6), implying that dextrose treatment could have a profound impact on the synthesis of HA during IC/BPS. However, additional *in vitro* experiments are required to verify.

As a result of the dextrose treatment in the regeneration of the uroepithelium glycosaminoglycan layer, it decreased the impulse threshold of the detrusor and pain response of nerve cells in the bladder submucosal layer (Lee et al., 2017). After the rats received the CYP injection, their urination frequency increased and the critical pressure threshold, the maximum bladder pressure, inter-contraction interval, and micturition volume of urination decreased (Figure 2). After saline or dextrose treatment, the frequent urination condition became prolonged. In addition, the pressure during urination of the CYP-induced rat was lower than that of the normal control group, which is different from the previous study (Liu et al., 2018). We suggest that the CYP-induced rat may cause serious damage to the detrusor muscle of the bladder. Therefore, even when the pressure threshold in the bladder does not reach the maximum pressure, the detrusor muscle will contract at the maximum value. In contrast, saline or dextrose treatment, the bladder maximum pressure and the frequency of urination improved, showing similar tendencies in the control group compared to the CYP group (Figure 2).

Further, our clinical study results showed that dextrose prolotherapy can improve the symptoms in patients with IC/BPS, which was indicated by significantly lessened nocturia and reduced cumulative treatment demand rate that implied the overall reduction in the number of people needing the treatment for a prolonged period. Most patients reported symptom improvement at least after the third cycle of the treatment. In addition, improved outcomes (GRA ≥2) at 4 weeks were reported in 12/29 patients (31.57%) receiving dextrose. Patients with dextrose treatment failure (GRA <1) had higher IPSS-V and IPSS-S values, as patients with dextrose treatment failure had a higher IPSS-TOTAL value (data not shown). Notably, decreased scores from questionnaires, including IPSS-TOTAL, IPSS-S, IPSS-V, VAS, ICSI, ICPI, and BSRS-5, at least after the first time of the treatment were also consistent with the report.

The mechanism of dextrose prolotherapy is still not definitively understood. Principally, the underlying theory of dextrose prolotherapy is to mimic the natural healing cascade of the body by stimulating an acute local inflammatory reaction at the injection site, thus activating the release of growth factors, fibroblast, and collagen deposition, which further lead to proliferation and improvement of new connective tissue, pain relief, and reduction of dysfunction (Hauser et al., 2011; Goswami, 2012). EGF, which is secreted by platelets, fibroblasts, and macrophages, plays a key role in wound healing as an important modulator of inflammatory response. It re-epithelializes and contributes to angiogenesis, fibroblast proliferation, formation of granulation tissue, and survival (Barrientos et al., 2008; Demidova-Rice et al., 2012). In our results (Table 4), the level of urinary EGF in IC/BPS patients significantly increased after 2 weeks of dextrose prolotherapy and remained high as time progressed. This suggested that dextrose was able to maintain a sustained level of EGF to continuously facilitate re-epithelialization in the proliferative cascade of tissue remodelling. Correspondingly, urinary HGF level was suppressed after dextrose intravesical injection. Studies have shown the anti-inflammatory mechanisms of HGF, wherein it reduces the production of pro-inflammatory cytokines mediated by macrophages and conversely enhances the secretion of adiponectin in adipocytes, thus further breaking the vicious cycle of inflammation mediated by the macrophages and adipocytes (Coudriet et al., 2010; Shimizu et al., 2012; Kusunoki et al., 2014). Furthermore, Kuo et al. (2013) demonstrated that the serum HGF elevation in patients with overactive bladder was associated with systemic neurotrophic factor elevation in the suburothelium of the bladder, which might play a role in the urgency and frequency sensory disorder (Kuo et al., 2013). Several studies have also demonstrated that serum HGF elevation has been identified in patients with varying types of disorders, such as obesity, metabolic syndrome, diabetes mellitus, multiple myeloma, gastric cancer, and bladder cancer, indicating that HGF levels were associated with disease progression (Oliveira et al., 2018; Wang et al., 2007; Corcoran et al., 2013; Sugimura et al., 1997). As HGF levels were notably decreased after weeks of treatment, it was considered that dextrose prolotherapy was likely to inhibit chronic inflammation and improve bladder function. Moreover, we found that PIGF-1 and VEGF-D levels were also remarkably decreased after following weeks of dextrose prolotherapy, which seemed to be associated with reduced HGF levels. When inducing angiogenesis, HGF was known to directly regulate angiogenic activity in vascular endothelial cells through the c-Met receptor and indirectly by facilitating vascular endothelial growth factor (VEGF) expression (Bussolino et al., 1992; Matsumura et al., 2013; Gerritsen et al., 2003). The human VEGF family includes VEGF-A, VEGF-B, VEGF-C, VEGF-D, and PIGF (Rissanen et al., 2003). We also found a strong correlation between the decrease in PIGF-1 level and the decrease of pain intensity on the VAS questionnaire (Figure 10). This indicated that lowering of PIGF-1 expression induced by dextrose prolotherapy may stimulate tissue repair and inhibit disease processes. Multiple cell types can express PIGF in different pathological conditions (Dewerchin and Carmeliet, 2012). Hence, inhibition of PIGF might reduce disease severity by improving vessel maturation and remodelling, decreasing atherogenesis and inflammatory cell infiltration, and attenuating cirrhotic disease (Roncal et al., 2010; Van Steenkiste et al., 2011).

Besides alteration of stimulating growth factors, we found that dextrose prolotherapy can affect the adaptive immune system in IC/BPS patients, which is primarily associated with particular subtypes of Th cells, such as Th1, Th2, Th17, and Treg. A significant upregulation of Treg type cytokine IL-10 in IC/BPS patients after dextrose prolotherapy demonstrated the proficiency of the treatment in attenuating the disease (Figure 11). Treg cells have an important role in tissue protection by limiting tissue damage, enforcing immune tolerance to self-tissues, and facilitating tissue repair (Arpaia et al., 2015; Alroqi and Chatila, 2016). They also maintain immune homeostasis and prevent autoimmunity through their immunosuppressive abilities (Alroqi and Chatila, 2016). IL-10 itself is referred to as the main anti-inflammatory and anti-fibrotic in active inflammation. It is believed to impede apoptotic signalling pathways, thereby limiting tissue damage and organ dysfunction after the injury (Krishnamurthy et al., 2009; Steen et al., 2020). In addition, dextrose is a key nutrient in the production of antimicrobial compounds (Brink et al., 2006), which is one of its probiotic properties. Probiotics can polarize the immune response toward Th1 profile response instead of Th2 response (Maldonado Galdeano et al., 2019). Accordingly, we suggest that dextrose prolotherapy could suppress IC/BPS sensitization by balancing Th1/Th2/Treg responses as a result of IL-10 production, which polarizes Th1 (IL-12p70 and IL-27) and alleviates Th2 (IL-13) responses. Moreover, when further compared to the healthy control (Figure 11; Table 4), our results demonstrated no significant difference in expressions of growth factors and cytokines displayed in IC/BPS patients after dextrose prolotherapy. This implies that this treatment is safe and a potential treatment strategy for IC/BPS management.

In this study, we used two different methods for dextrose administration in animal and human studies, including intravesical instillation and intravesical injection, respectively. Intravesical injection of dextrose is a normal method used in clinical. However, as pointed out by previous studies that IC/BPS improvement is related to the ECM repair process, hence, we developed a rat model of IC/BPS and performed intravesical instillation of dextrose solution to gather more insights into IC/BPS pathologies. According to the findings of animal study, we found that intravesical instillation of dextrose provided beneficial effects, and in the future, this method is potentially applicable for human trials. In addition, a larger number of patients for intravesical injection of dextrose study is also essential for confirming the efficacy of the treatment.

One of the novelties of this study is that we provide insights and evidence for translational medicine. We combined CYP-induced IC/BPS rat model to study the possible pathogenic mechanism of IC/BPS and found it to be related to the HA biosynthesis dysfunction of the urothelial bladder tissue. Utilizing the dextrose treatment to increase ECM and HA synthesis of the bladder, the results reveal the pathogenic mechanism of IC/BPS and provide reliable clinical treatment strategies, supported by the effectiveness of treatment in this animal study. In addition, the clinical trial investigates the therapeutic efficiency of dextrose. According to the expression of growth factors in IC patient’s urine, dextrose treatment could directly affect the regulation of the ECM synthesis and production of the bladder urothelium.

There are some limitations to our present study. We eventually enrolled a small number of patients, which may have limited the collection of questionnaires, because the patients left the trial early due to restored bladder function or relief of pain and refusing back to the hospital following the rapid rising COVID-19 cases in Taiwan. In contrast, we unexpectedly found that the dextrose treatment allowed patients to obtain a comfortable QoL earlier. Another limitation was this cohort study was designed to compare the difference in bladder function of IC/BPS patients before and after the dextrose treatment. There was no parallel placebo group. We did not collect biopsy from the bladder of IC/BPS patients to avoid further urothelium damage. Thus, we have limited information on pathological regulation. Further well-designed randomized trials to confirm the results of dextrose prolotherapy for IC/BPS are needed.

Summarily, our animal study results demonstrated that dextrose prolotherapy improved bladder hyperactivity and damage by increasing the expression of HA receptors and HA synthase as well as undergoing cell proliferation and differentiation in response to IC/BPS injury. In clinical results, dextrose prolotherapy exhibited a novel and safe treatment for refractory IC/BPS that can affect the adaptive immune system which inhibits chronic inflammation and improves bladder function. The results also demonstrated that this treatment can improve the symptoms in IC/BPS patients, and it was beneficial when compared to the patients’ previous 6-month intravesical instillations of hyaluronic acid and/or Botox which had no improved condition. Hence, our study suggests that dextrose prolotherapy appears to significantly relieve the symptoms of IC/BPS patients and can be considered a potential treatment strategy for IC/BPS management.

## Data Availability

The original contributions presented in the study are included in the article/supplementary material, further inquiries can be directed to the corresponding author.

## References

[B1] Ahmad I. Sarath Krishna N. Meddings R. N. (2008). Sequential Hydrodistension and Intravesical Instillation of Hyaluronic Acid under General Anaesthesia for Treatment of Refractory Interstitial Cystitis: a Pilot Study. Int. Urogynecol. J. Pelvic Floor Dysfunct. 19 (4), 543–546. 10.1007/s00192-007-0443-4 17874027

[B2] Alroqi F. J. Chatila T. A. (2016). T Regulatory Cell Biology in Health and Disease. Curr. Allergy Asthma Rep. 16 (4), 27. 10.1007/s11882-016-0606-9 26922942 PMC5218767

[B3] Arpaia N. Green J. A. Moltedo B. Arvey A. Hemmers S. Yuan S. (2015). A Distinct Function of Regulatory T Cells in Tissue protection. Cell 162 (5), 1078–1089. 10.1016/j.cell.2015.08.021 26317471 PMC4603556

[B4] Augé C. Gamé X. Vergnolle N. Lluel P. Chabot S. (2020). Characterization and Validation of a Chronic Model of Cyclophosphamide-Induced Interstitial Cystitis/bladder Pain Syndrome in Rats. Front. Pharmacol. 11, 1305. 10.3389/fphar.2020.01305 32982733 PMC7485435

[B5] Barrientos S. Stojadinovic O. Golinko M. S. Brem H. Tomic-Canic M. (2008). Growth Factors and Cytokines in Wound Healing. Wound Repair Regen. 16 (5), 585–601. 10.1111/j.1524-475X.2008.00410.x 19128254

[B6] Birder L. Andersson K.-E. (2018). Animal Modelling of Interstitial Cystitis/bladder Pain Syndrome. Int. Neurourol J. 22 (Suppl. 1), S3–S9. 10.5213/inj.1835062.531 29385788 PMC5798638

[B7] Brink M. Todorov S. D. Martin J. H. Senekal M. Dicks L. M. (2006). The Effect of Prebiotics on Production of Antimicrobial Compounds, Resistance to Growth at Low pH and in the Presence of Bile, and Adhesion of Probiotic Cells to Intestinal Mucus. J. Appl. Microbiol. 100 (4), 813–820. 10.1111/j.1365-2672.2006.02859.x 16553737

[B8] Bussolino F. Di Renzo M. F. Ziche M. Bocchietto E. Olivero M. Naldini L. (1992). Hepatocyte Growth Factor Is a Potent Angiogenic Factor Which Stimulates Endothelial Cell Motility and Growth. J. Cel Biol 119 (3), 629–641. 10.1083/jcb.119.3.629 PMC2289675 1383237

[B9] Cervigni M. Natale F. Nasta L. Padoa A. Voi R. L. Porru D. (2008). A Combined Intravesical Therapy with Hyaluronic Acid and Chondroitin for Refractory Painful Bladder Syndrome/interstitial Cystitis. Int. Urogynecol. J. Pelvic Floor Dysfunct. 19 (7), 943–947. 10.1007/s00192-008-0572-4 18338095

[B10] Chancellor M. B. Yoshimura N. (2004). Treatment of Interstitial Cystitis. Urology 63 (4 Pt 1), 85–92. 10.1016/j.urology.2003.10.034 15013658

[B11] Chen H. C. Wu C. H. Lee Y. J. Liao S. C. Lee M. B. (2005). Validity of the Five-Item Brief Symptom Rating Scale Among Subjects Admitted for General Health Screening. J. Formos. Med. Assoc. 104 (11), 824–829. 16496062

[B12] Chen W. Y. Abatangelo G. (1999). Functions of Hyaluronan in Wound Repair. Wound Repair Regen. 7 (2), 79–89. 10.1046/j.1524-475x.1999.00079.x 10231509

[B13] Chung S. J. Yang D.-O. Yu H. S. Park K. (2019). Development and Validation of the Korean Version of the Female Sexual Function Index-5 (FSFI-5). Korean J. Sex. Health 3, 3–9. 10.34224/kjsh.2019.3.1.3

[B14] Corcoran A. T. Yoshimura N. Tyagi V. Jacobs B. Leng W. Tyagi P. (2013). Mapping the Cytokine Profile of Painful Bladder Syndrome/interstitial Cystitis in Human Bladder and Urine Specimens. World J. Urol. 31 (1), 241–246. 10.1007/s00345-012-0852-y 22441309 PMC3674577

[B15] Coudriet G. M. He J. Trucco M. Mars W. M. Piganelli J. D. (2010). Hepatocyte Growth Factor Modulates Interleukin-6 Production in Bone Marrow Derived Macrophages: Implications for Inflammatory Mediated Diseases. PLoS One 5 (11), e15384. 10.1371/journal.pone.0015384 21072211 PMC2970559

[B16] Croce M. A. Dyne K. Boraldi F. Quaglino D. Jr Cetta G. Tiozzo R. (2001). Hyaluronan Affects Protein and Collagen Synthesis by *In Vitro* Human Skin Fibroblasts. Tissue Cell 33 (4), 326–331. 10.1054/tice.2001.0180 11521947

[B17] Demidova-Rice T. N. Hamblin M. R. Herman I. M. (2012). Acute and Impaired Wound Healing: Pathophysiology and Current Methods for Drug Delivery, Part 2: Role of Growth Factors in normal and Pathological Wound Healing: Therapeutic Potential and Methods of Delivery. Adv. Skin Wound Care 25 (8), 349–370. 10.1097/01.ASW.0000418541.31366.a3 22820962 PMC3426909

[B18] Dewerchin M. Carmeliet P. (2012). PlGF: A Multitasking Cytokine with Disease-Restricted Activity. Cold Spring Harb Perspect. Med. 2 (8), a011056. 10.1101/cshperspect.a011056 22908198 PMC3405829

[B19] Garzon S. Laganà A. S. Casarin J. Raffaelli R. Cromi A. Sturla D. (2020). An Update on Treatment Options for Interstitial Cystitis. Prz Menopauzalny 19 (1), 35–43. 10.5114/pm.2020.95334 32699542 PMC7258371

[B20] Gerritsen M. E. Tomlinson J. E. Zlot C. Ziman M. Hwang S. (2003). Using Gene Expression Profiling to Identify the Molecular Basis of the Synergistic Actions of Hepatocyte Growth Factor and Vascular Endothelial Growth Factor in Human Endothelial Cells. Br. J. Pharmacol. 140 (4), 595–610. 10.1038/sj.bjp.0705494 14504135 PMC1574080

[B21] Goswami A. (2012). Prolotherapy. J. Pain Palliat. Care Pharmacother. 26 (4), 376–378. 10.3109/15360288.2012.734900 23216178

[B22] Hanno P. M. (1997). Analysis of Long-Term Elmiron Therapy for Interstitial Cystitis. Urology 49, 93–99. 10.1016/S0090-4295(97)00179-9 9146008

[B23] Hauser R. A. Hauser M. A. Baird N. M. (2011). Evidence-based Use of Dextrose Prolotherapy for Musculoskeletal Pain: a Scientific Literature Review. J. Prolotherapy 3, 765.

[B24] Hauser R. A. Lackner J. B. Steilen-Matias D. Harris D. K. (2016). A Systematic Review of Dextrose Prolotherapy for Chronic Musculoskeletal Pain. Clin. Med. Insights Arthritis Musculoskelet. Disord. 9, 139–159. 10.4137/CMAMD.S39160 27429562 PMC4938120

[B25] Iavazzo C. Athanasiou S. Pitsouni E. Falagas M. E. (2007). Hyaluronic Acid: an Effective Alternative Treatment of Interstitial Cystitis, Recurrent Urinary Tract Infections, and Hemorrhagic Cystitis? Eur. Urol. 51 (6), 1534–1540. 10.1016/j.eururo.2007.03.020 17383810

[B26] Kaya G. Rodriguez I. Jorcano J. L. Vassalli P. Stamenkovic I. (1997). Selective Suppression of CD44 in Keratinocytes of Mice Bearing an Antisense CD44 Transgene Driven by a Tissue-specific Promoter Disrupts Hyaluronate Metabolism in the Skin and Impairs Keratinocyte Proliferation. Genes Dev. 11 (8), 996–1007. 10.1101/gad.11.8.996 9136928

[B27] Krishnamurthy P. Rajasingh J. Lambers E. Qin G. Losordo D. W. Kishore R. (2009). IL-10 Inhibits Inflammation and Attenuates Left Ventricular Remodeling after Myocardial Infarction via Activation of STAT3 and Suppression of HuR. Circ. Res. 104 (2), e9–18. 10.1161/CIRCRESAHA.108.188243 19096025 PMC2774810

[B28] Kuo H.-C. Liu H.-T. Shie J.-H. (2013). Potential Urine and Serum Biomarkers for Patients with Overactive Bladder and Interstitial Cystitis/bladder Pain Syndrome. Tzu Chi Med. J. 25, 13–18. 10.1016/j.tcmj.2012.10.005

[B29] Kusunoki H. Taniyama Y. Otsu R. Rakugi H. Morishita R. (2014). Anti-inflammatory Effects of Hepatocyte Growth Factor on the Vicious Cycle of Macrophages and Adipocytes. Hypertens. Res. 37 (6), 500–506. 10.1038/hr.2014.41 24621470

[B30] Lee Y. L. Lin K. L. Chuang S. M. Lee Y. C. Lu M. C. Wu B. N. (2017). Elucidating Mechanisms of Bladder Repair after Hyaluronan Instillation in Ketamine-Induced Ulcerative Cystitis in Animal Model. Am. J. Pathol. 187 (9), 1945–1959. 10.1016/j.ajpath.2017.06.004 28826558

[B31] Liu Q. Sun B. Zhao J. Wang Q. An F. Hu X. (2018). Increased Piezo1 Channel Activity in Interstitial Cajal-like Cells Induces Bladder Hyperactivity by Functionally Interacting with NCX1 in Rats with Cyclophosphamide-Induced Cystitis. Exp. Mol. Med. 50 (5), 1–16. 10.1038/s12276-018-0088-z PMC5938236 29735991

[B32] Lubeck D. P. Whitmore K. Sant G. R. Alvarez-Horine S. Lai C. (2001). Psychometric Validation of the O'leary-Sant Interstitial Cystitis Symptom index in a Clinical Trial of Pentosan Polysulfate Sodium. Urology 57 (6 Suppl. 1), 62–66. 10.1016/s0090-4295(01)01126-8 11378052

[B33] Maldonado Galdeano C. Cazorla S. I. Lemme Dumit J. M. Vélez E. Perdigón G. (2019). Beneficial Effects of Probiotic Consumption on the Immune System. Ann. Nutr. Metab. 74 (2), 115–124. 10.1159/000496426 30673668

[B34] Matsumura A. Kubota T. Taiyoh H. Fujiwara H. Okamoto K. Ichikawa D. (2013). HGF Regulates VEGF Expression via the C-Met Receptor Downstream Pathways, Pi3k/akt, Mapk and Stat3, in Ct26 Murine Cells. Int. J. Oncol. 42 (2), 535–542. 10.3892/ijo.2012.1728 23233163

[B35] Meng E. Chang H. Y. Chang S. Y. Sun G. H. Yu D. S. Cha T. L. (2011). Involvement of Purinergic Neurotransmission in Ketamine Induced Bladder Dysfunction. J. Urol. 186 (3), 1134–1141. 10.1016/j.juro.2011.04.102 21784472

[B36] Mondalek F. G. Fung K. M. Yang Q. Wu W. Lu W. Palmer B. W. (2015). Temporal Expression of Hyaluronic Acid and Hyaluronic Acid Receptors in a Porcine Small Intestinal Submucosa-Augmented Rat Bladder Regeneration Model. World J. Urol. 33 (8), 1119–1128. 10.1007/s00345-014-1403-5 25253654 PMC4699683

[B37] Nair L. S. (2011). Prolotherapy for Tissue Repair. Transl Res. 158 (3), 129–131. 10.1016/j.trsl.2011.05.001 21867977

[B38] Nickel J. C. (2004). Interstitial Cystitis: A Chronic Pelvic Pain Syndrome. Med. Clin. North. Am. 88 (2), 467–xii. 10.1016/S0025-7125(03)00151-2 15049588

[B39] Oliveira A. G. Araújo T. G. Carvalho B. d. M. Rocha G. Z. Santos A. Saad M. J. A. (2018). The Role of Hepatocyte Growth Factor (HGF) in Insulin Resistance and Diabetes. Front. Endocrinol. 9, 503. 10.3389/fendo.2018.00503 PMC6125308 30214428

[B40] Rabago D. Reeves K. D. Doherty M. P. Fleck M. (2019). Prolotherapy for Musculoskeletal Pain and Disability in Low- and Middle-Income Countries. Phys. Med. Rehabil. Clin. N. Am. 30 (4), 775–786. 10.1016/j.pmr.2019.07.003 31563169

[B41] Reitinger S. Lepperdinger G. (2013). Hyaluronan, a Ready Choice to Fuel Regeneration: a Mini-Review. Gerontology 59 (1), 71–76. 10.1159/000342200 23006468

[B42] Rissanen T. T. Markkanen J. E. Gruchala M. Heikura T. Puranen A. Kettunen M. I. (2003). VEGF-D Is the Strongest Angiogenic and Lymphangiogenic Effector Among VEGFS Delivered into Skeletal Muscle via Adenoviruses. Circ. Res. 92 (10), 1098–1106. 10.1161/01.RES.0000073584.46059.E3 12714562

[B43] Roncal C. Buysschaert I. Gerdes N. Georgiadou M. Ovchinnikova O. Fischer C. (2010). Short-term Delivery of Anti-PLGF Antibody Delays Progression of Atherosclerotic Plaques to Vulnerable Lesions. Cardiovasc. Res. 86 (1), 29–36. 10.1093/cvr/cvp380 19952000

[B44] Sand P. K. Kaufman D. M. Evans R. J. Zhang H. F. Alan Fisher D. L. Nickel J. C. (2008). Association between Response to Pentosan Polysulfate Sodium Therapy for Interstitial Cystitis and Patient Questionnaire-Based Treatment Satisfaction. Curr. Med. Res. Opin. 24 (8), 2259–2264. 10.1185/03007990802240727 18582395

[B45] Shimizu K. Taniyama Y. Sanada F. Azuma J. Iwabayashi M. Iekushi K. (2012). Hepatocyte Growth Factor Inhibits Lipopolysaccharide-Induced Oxidative Stress via Epithelial Growth Factor Receptor Degradation. Arterioscler Thromb. Vasc. Biol. 32 (11), 2687–2693. 10.1161/ATVBAHA.112.300041 22936342

[B46] Sit R. W. Chung V. Ch. Reeves K. D. Rabago D. Chan K. K. Chan D. C. (2016). Hypertonic Dextrose Injections (Prolotherapy) in the Treatment of Symptomatic Knee Osteoarthritis: A Systematic Review and Meta-Analysis. Sci. Rep. 6, 25247. 10.1038/srep25247 27146849 PMC4857084

[B47] Steen E. H. Wang X. Balaji S. Butte M. J. Bollyky P. L. Keswani S. G. (2020). The Role of the Anti-inflammatory Cytokine Interleukin-10 in Tissue Fibrosis. Adv. Wound Care (New Rochelle) 9 (4), 184–198. 10.1089/wound.2019.1032 32117582 PMC7047112

[B48] Sugimura K. Lee C. C. Kim T. Goto T. Kasai S. Harimoto K. (1997). Production of Hepatocyte Growth Factor Is Increased in Chronic Renal Failure. Nephron 75 (1), 7–12. 10.1159/000189492 9031263

[B49] Tsai S. W. Hsu Y. J. Lee M. C. Huang H. E. Huang C. C. Tung Y. T. (2018). Effects of Dextrose Prolotherapy on Contusion-Induced Muscle Injuries in Mice. Int. J. Med. Sci. 15 (11), 1251–1259. 10.7150/ijms.24170 30123064 PMC6097270

[B50] Van Steenkiste C. Ribera J. Geerts A. Pauta M. Tugues S. Casteleyn C. (2011). Inhibition of Placental Growth Factor Activity Reduces the Severity of Fibrosis, Inflammation, and portal Hypertension in Cirrhotic Mice. Hepatology 53 (5), 1629–1640. 10.1002/hep.24238 21520176

[B51] Vittinghoff E. Glidden D. V. Shiboski S. C. McCulloch C. E. (2005). Regression Methods in Biostatistics: Linear, Logistic, Survival, and Repeated Measures Models. New York: Springer Publishing Co., 266–289.

[B52] Vora A. Borg-Stein J. Nguyen R. T. (2012). Regenerative Injection Therapy for Osteoarthritis: Fundamental Concepts and Evidence-Based Review. PM R. 4 (5 Suppl. l), S104–S109. 10.1016/j.pmrj.2012.02.005 22632688

[B53] Wang P. Nishitani M. A. Tanimoto S. Kishimoto T. Fukumori T. Takahashi M. (2007). Bladder Cancer Cell Invasion Is Enhanced by Cross-Talk with Fibroblasts through Hepatocyte Growth Factor. Urology 69 (4), 780–784. 10.1016/j.urology.2007.01.063 17445681

[B54] Zaman A. Cui Z. Foley J. P. Zhao H. Grimm P. C. Delisser H. M. (2005). Expression and Role of the Hyaluronan Receptor RHAMM in Inflammation after Bleomycin Injury. Am. J. Respir. Cel Mol Biol 33 (5), 447–454. 10.1165/rcmb.2004-0333OC PMC2715352 16037485

